# Supplementary material for: Spike-Dependent Dynamic Partitioning of the Locus Coeruleus Network through Noradrenergic Volume Release in a Simulation of the Nucleus Core
Source: Brain Sci. 2022 Jun 1;12(6):728. doi: 10.3390/brainsci12060728 (PMC9221380; doi:10.3390/brainsci12060728)
Supplement: Supplementary file 1 [file brainsci-12-00728-s001.zip › brainsci-1596062-supplementary.pdf]

Supplementary File

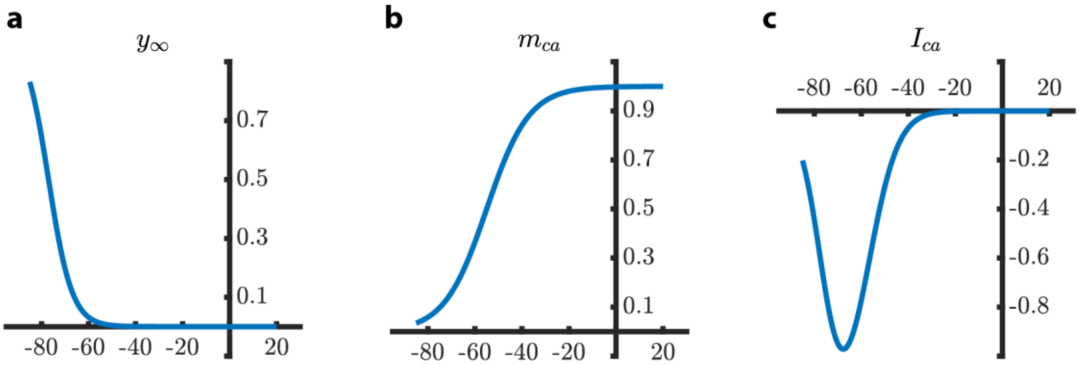

Figure S1. The calcium current is responsible for the spontaneous spiking of the cell. It is unclear in Alvarez et al if this is a transient conductance (with activation and inactivation gates) or a persistent conductance (with two kinds of activation gates). If we take the formulas as given, The 'fast' gating variable mCa opens with membrane depolarization. Specifically, it approaches unity for membrane potentials above −40 mV (see Supp Figure S1). The 'slow' gating variable y∞ closes with membrane depolarization. Specifically, it approaches zero for membrane potentials above −60 mV . In the same regime, the characteristic time to approaches 20 ms. Taken together, this current is active in a window between −80mV and −40mV , being strongest around −60mV . As a result, this current can cause a slow Ca-spike, on which multiple fast Na-spikes can occur. Activation variables for y∞ (left) and m_ca (middle) and I_ca (right) for calcium current, as a function of membrane voltage, for the formulas as given by Alvarez et al.

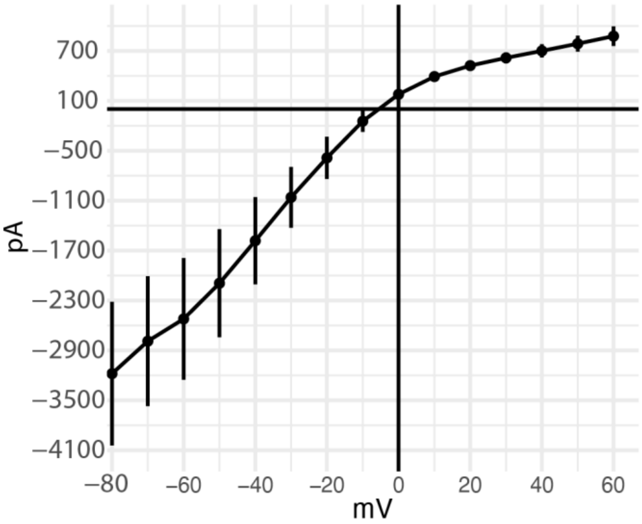

Figure S2. Inward-rectification presented in a current-voltage plot derived from HEK293 that heterologus express GIRK2 channels. GIRK time course are used in equation 13.

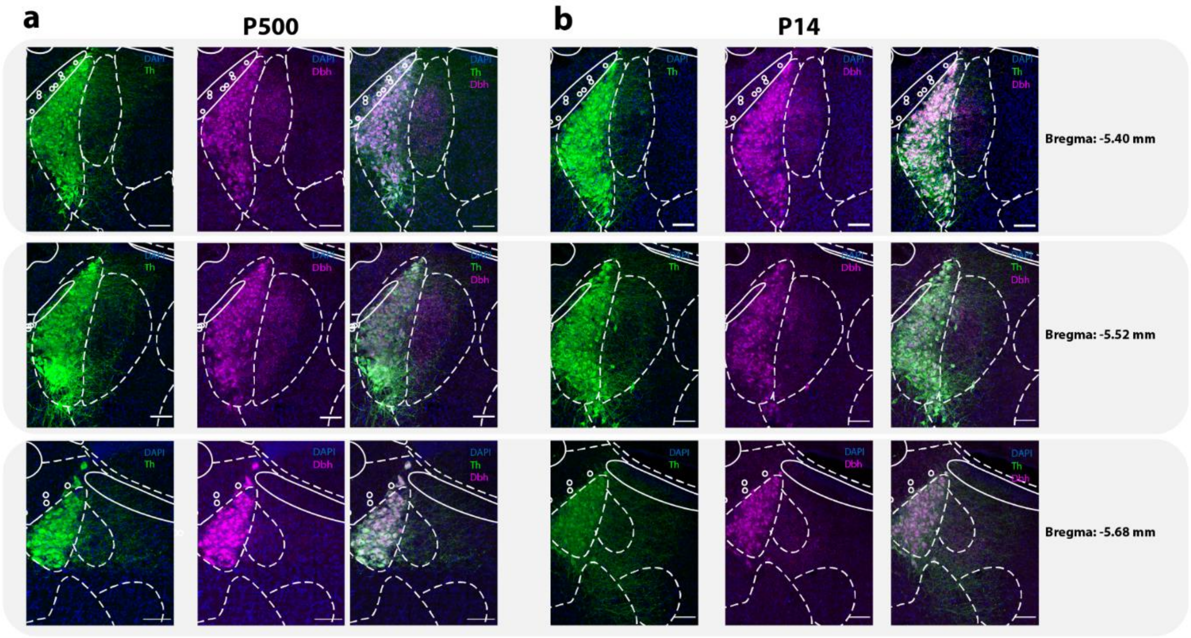

Figure S3. Confocal images of immunostained brainstem slices against tyrosine hydroxylase (TH / green) and dopamin-beta-hydroxylase (DbH / magenta) at three different position from bregma: - 5.40 , - 5.52 and -5.68mm. **(a)** Shows LCs slices from an old animal (P500), while **(b)** overviews LC slices from a young animal (P14). Somatic as well as dendritic compartments exhibit different expression levels for TH and DbH within the LC topography.

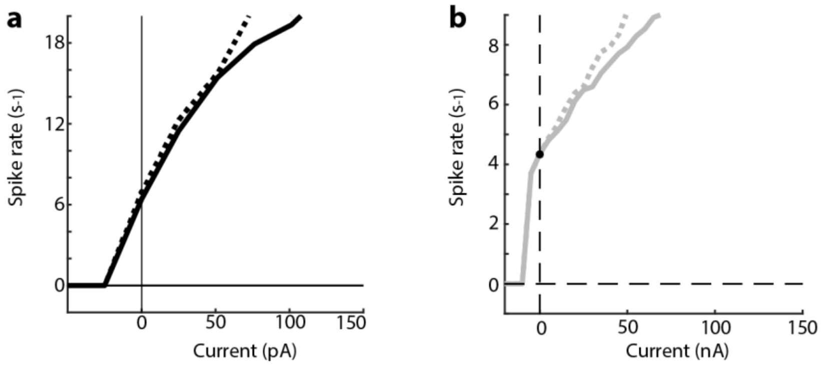

Figure S4. Spike rate dependence on 300-ms current-injection steps separated for early spikes (spike 1 and 2 / dotted line) versus later spikes (> spike 2 / full line). **(a)** FI Curve of an experimental neuron. It is firing at almost 6.1Hz at spontaneous state. **(b)** FI curve of a simulated neuron. It is firing at almost 4.5Hz at spontaneous state.

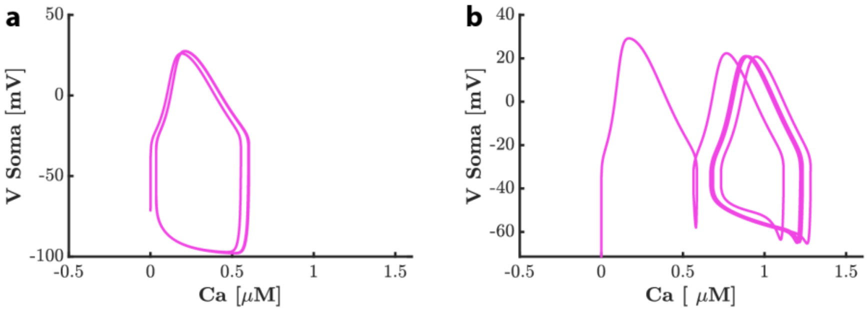

Figure S5. Action potential triggers calcium accumulation in the cell which facilitates the release of neurotransmitters along with many other calcium-triggered events. When the simulated neuron is firing at 4Hz, a small amount of calcium is accumulated and

equibrilates at constant level as shown in fig 3 **(a)**. When the same neuron is firing at 20Hz, a higher level of calcium is accumulated after several action potentials **(b)**. .

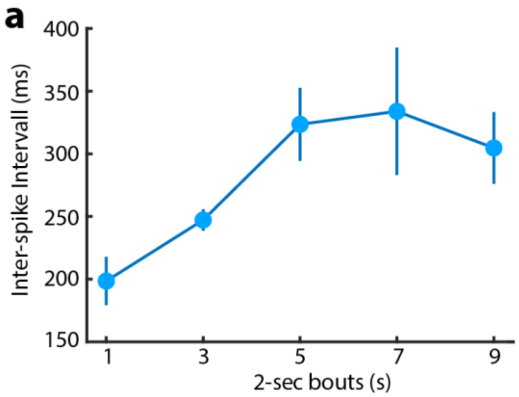

Figure S6. Effect of extracellular NE that diffuses freely in the extracellular space. The released NE binds to a receptor site in a nearby neuron and triggers opening of GIRK. Unlike synaptic inhibition, inhibition by diffusion is delayed in nature as it takes time to reach the binding site when diffused in extracellular space. Here ISI (binned 2s) are calculated for neuron N2 in figure 5. Inhibition slowly builds up (1s-7s) after N1 releases NE during several intervals which leads to a significantly longer ISI in N3. N3 slowly gets back to the steady state after N1 stops releasing NE(9s). .
